# My Throat is Itchy! An In-situ Simulation for Interprofessional Healthcare Education

**DOI:** 10.7759/cureus.4366

**Published:** 2019-04-02

**Authors:** Jennifer Dale-Tam, Kelly McBride

**Affiliations:** 1 Medical Education and Simulation, The Ottawa Hospital, Ottawa, CAN

**Keywords:** simulation, inter-professional education, latent safety threats, anaphylaxis, in-situ simulation, nursing, physician

## Abstract

In-situ simulation occurs in the clinical environment. This allows healthcare providers greater access to the educational session while providing the opportunity to test systems or protocols in place. Anaphylaxis is a rare and life-threatening event. As such, many healthcare providers are uncomfortable managing it. The use of simulation as an educational methodology allows the learners to practice rare, high-risk scenarios in a low-risk environment. There is no negative impact to an actual patient when an in-situ simulation education session is provided. Usually there are positive results due to increased staff awareness and improved process.

In the spring of 2015, stakeholders at the outpatient antibiotic therapy program (OPAT) at The Ottawa Hospital (TOH) approached the nurse educator team to develop an education session around anaphylaxis management. The nurse educators chose to design and implement an in-situ simulation scenario involving the inter-professional clinic team. Through the use of inter-professional in-situ simulation the team was able to clarify roles, identify equipment issues and rectify those issues as this technical report describes.

## Introduction

Anaphylaxis is a life-threatening condition that often goes unrecognized by healthcare professionals [1]. Anaphylaxis is defined as a systemic response of urticaria, angioedema, respiratory depression, vasodilatation which can lead to cardiovascular and respiratory collapse resulting in death [2]. Urgent recognition and treatment is paramount to reduce morbidity and mortality of patients. One of the most common triggers for anaphylaxis is medications, specifically antibiotics. From 1999 to 2010, in the United States, drugs accounted for 58% of anaphylactic fatalities [1]. For these reasons many healthcare organizations administer first dose intravenous (IV) antibiotics in supervised medical settings [3].

One of the main underpinnings of simulation-based education is the safety of patients and participants. Two in-situ simulation (ISS) scenarios were developed and implemented for inter-professional healthcare education at the request of the leadership team of the outpatient antibiotic therapy program (OPAT) clinic at The Ottawa Hospital (TOH). ISS was chosen over theatre-based simulation as it provides opportunity to practice in the real clinical environment allowing for many members of the healthcare team to attend. ISS can also audit the healthcare system [4].

Scenario development occurred in the spring of 2015 with implementation in May and June of the same year after discussion with stakeholders. In-situ sessions were planned to optimize attendance and provide minimal disruption to clinic function. The sessions were attended by Registered Practical Nurses (RPNs), infectious disease residents and physicians. A total of four sessions occurred consisting of a five-minute pre-brief, seven- to ten-minute scenario followed by a 15- to 20-minute debrief. The scenario presented in this report is of a patient with antibiotics infusing via IV. The simulation manikin and technician were booked through the TOH Safe Patient Simulation Program.

Learning objectives

During the anaphylaxis ISS the RPN will:

1. identify patient is having an anaphylactic reaction;

2. call physician for help;

3. stop the antibiotic infusion;

4. start oxygen as per TOH institutional policy and directives; and

5. communicate with physician(s) using closed loop communication.

During the anaphylaxis ISS the physician will:

1. initiate the TOH anaphylaxis algorithm;

2. administer intramuscular (IM) epinephrine; and

3. communicate with RPN(s) using closed loop communication.

## Technical report

Case summary

Christina Smith is a 56-year-old female coming into clinic for treatment of a wound infection on her right leg. She has a remote allergy to penicillin from childhood with cutaneous reaction and is unclear of current allergy status. She is receiving her first dose of IV piperacillin/tazobactam (Pip/Tazo) which has been infusing for 10 minutes. The scenario starts with Christina calling the RPN into the clinic treatment room complaining of an itchy throat. The RPN should assess the patient, identify a possible anaphylactic reaction, stop the antibiotic, call the physician and start oxygen. The physician should initiate the anaphylactic algorithm and give IM epinephrine.

Personnel

· Simulation Instructor

· Simulation technician

· Anaphylaxis content expert-if required

Learners

· Primary RPN

· Secondary RPN

· Infectious disease physician or resident

· Observers

Learner preparation

A pre-brief was provided to all participants addressing scenario confidentiality, purpose of the scenario for formative learning, orientation to the manikin and equipment and the simulation basic assumption that learners are there to do their best.

Healthcare providers are given standard high-level objectives, which are used across all simulations associated with the surgery nursing education program, during pre-briefing:

You are to communicate with the patient, your colleagues and inter-professional team as appropriate;

You are to assess the patient, identify any issues and intervene accordingly.

Primary RPN: you are working in the first dose antibiotic clinic where Christina Smith is receiving her first dose of IV Pip/Tazo 3.375 g for a wound infection on her right leg. The infusion was started 10 minutes ago. She has called you back into the room as she is not feeling well.

Secondary RPN: you come into the room when called and assist where needed.

Physician: you are documenting in the clinic office and are called for assistance in one of the clinic rooms.

Observers: you are to observe the simulation, take notes and prepare to participate in the debrief.

Set up

Refer to Appendix A for equipment list.

The scenario takes place in a treatment room of an ambulatory care clinic during regular office hours associated with a tertiary care hospital.

A high-fidelity manikin dressed in a t-shirt and pants in an outpatient chair with a primary IV infusing of normal saline and a secondary IV line with Pip/Tazo 3.375 g infusing via an IV infusion pump. A vital signs monitor (heart rate, blood pressure, temperature and pulse oximetry) is available in the room. An oxygen flow meter is available in the room. None are attached to the manikin.

The standard nursing station within the clinic is available. A fully stocked cardiac arrest cart with defibrillator is available in the clinic. A stocked emergency drug box (Appendix B) is kept near the treatment room. Nasal prongs and related oxygen masks are available in the stock room of the clinic along with IV tubing and various infusion bags.

Scenario progression

The optimal scenario progression (Table 1) with expected interventions by the nurses and physicians is described below. Instructor notes along with triggers to advance the scenario (Table 2) when healthcare providers are not progressing towards the learning objectives of the simulation are also listed.

**Table 1:** Scenario Progression. BP: Blood pressure; HR: Heart rate; IM: Intramuscular; IV: Intravenous; MD: Medical doctor; meds: medications; MERT: Medical emergency response team; mcg: micrograms; mg: milligrams; mL/hr: milliliters per hour; NS: Normal saline; O_2_: Oxygen; pip/tazo: piperacillin tazobactam; RA: Room air; RPN: Registered practical nurse; RR: Respiratory rate; SBAR: Situation background assessment recommendation; 1^st^: First; 2^nd^: Second; %: Percent.

Timing (approximate)	Manikin Programming and Actions	Expected Interventions: RPNs	Expected Interventions: Physician
Start	Sitting in Chair HR 95, BP 130/80, RR 18, O_2_ saturation 97%, “I am itchy all over.” "Look at the rash on my neck."	1^st^ RPN enters room. Introduces self to patient. Assesses Christina. Stop the pip/tazo infusion. Switch to primary infusion of NS at 100 mL/hr.	
One minute	Same “My throat and eyes, are itchy.” “My mouth is tingling.”	Call 2^nd^ RPN for assistance. 1^st^ RPN stays with patient and completes a set of vital signs. 2^nd^ RPN contacts physician. 1^st ^RPN gives report to MD using SBAR.	Physician arrives in room. Receives report from 1^st^ RPN. Assesses patient.
Two minutes	Same	2^nd^ RPN grabs the emergency drug box. 1^st^ RPN starts the IV bolus as per physician orders.	Identifies patient is having an anaphylactic reaction. Initiates anaphylaxis protocol.
Three minutes	Same	RPN states “I cannot give IV epinephrine; I can give it IM.”	Orders epinephrine 300 mcg IV. Changes epinephrine order to 300 mcg IM. Considers 2^nd^ line meds: ranitidine 50 mg IV, diphenhydramine 50 mg IV, methylprednisolone 125 mg IV.
Four minutes	HR 120, BP 90/40, RR 25, O_2_ saturation 88% on RA. Coughing	1^st^ RPN gives 300 mcg epinephrine; communicates to MD “300 mcg of epinephrine IM given”. 2^nd^ RPN completes a second set of vital signs and communicates results to MD.	"Thank you for the new set of vital signs." Orders ranitidine 50 mg IV, diphenhydramine 50 mg IV, methylprednisolone 125 mg IV
Five minutes	Same	1^st^ RPN starts O_2 _via face mask. 2^nd^ RPN prepares ranitidine 50 mg IV, diphenhydramine 50 mg IV, methylprednisolone 125 mg IV	“Please call the medical emergency response team”
Six minutes	Same	1^st^ RPN gives diphenhydramine 50 mg IV via secondary IV set. 2^nd^ RPN calls MERT; tells MD MERT was called	“Thank you for calling.”
Seven minutes	Continues coughing.	1^st^ RPN “the algorithm says you can give the epinephrine every 3 to 5 minutes. We gave the first dose just over 3 minutes ago”.	Considers when to give another dose of IM epinephrine; “nurse when can we give another dose of epinephrine?”
Eight minutes	Same	MERT arrives. 1^st^ RPN is present when report given to MERT team by MD. 2^nd^ RPN monitors patient.	Gives report to MERT using SBAR.

**Table 2:** Prompts to Advance Scenario. IV: Intravenous; MD: Medical doctor; MERT: Medical emergency response team; O_2_: Oxygen; RPN: Registered practical nurse; &: and; %: percent.

Time (approximate)	Actions	Triggers
0-2 minutes	RPN does not stop antibiotic infusion.	Christina: “My throat is really, REALLY, itchy and so is my arm!”
2-4 minutes	RPN gives epinephrine IV.	Christina goes into cardiac arrest.
4-6 minutes	RPN does not put on O_2_.	Decrease O_2_ saturation to 85% & becomes more stridorous.
6-8 minutes	MD does not call MERT.	Christina becomes more unstable.

Debriefing

Debriefing following a simulation scenario allows learners to decompress and reflect on what occurred during the scenario in the continuous learning cycle [5]. It is advantageous to have the debriefing conversation facilitated by an experienced simulation instructor with knowledge of anaphylaxis management to streamline the number of individuals in the debrief. If the instructor does not have experience in anaphylaxis management then a content expert should be present to close knowledge and performance gaps as it is a high-risk medical condition that requires appropriate medical management. Debriefing with a thorough framework provides structure and flow to the debriefing. The Promoting Excellence and Reflective Learning in Simulation (PEARLS) framework is used for all debriefing sessions within the surgery nursing education program at TOH [6].

## Discussion

Simulations are moving out of the brick and mortar simulation centers into the clinical environment with the implementation of ISSs over the last five years. These provide greater contextual realism to the scenario and increase access for frontline healthcare providers to participate in this learning experience. ISSs allow for assessment of knowledge of the individual and team along with auditing of the system as a whole, which can lead to the identification of latent safety threats (LSTs) [4]. During the anaphylaxis simulations numerous LSTs were identified and discussed during the debrief. These discussions informed several modifications that were made to the clinical environment to optimize patient safety.

Role clarity regarding medication administration

The physicians were unsure of the scope of practice of RPNs versus registered nurses (RNs). Many of the physicians practiced with RNs in the inpatient environment and worked with RPNs in the outpatient clinic. Though both are generally referred to as a nurse in the lexicon of healthcare providers, the regulated abilities are quite different when it comes to medication administration. The main difference being that RNs can give direct IV medications and RPNs can only give IV medications via the secondary route, also known as piggyback. This provided clarity regarding second line medications when managing anaphylaxis. The first line medication of epinephrine is ordered as IV or IM. The use of IM epinephrine as being within the scope for RPNs to administer was reviewed within the debrief.

Emergency drug box contents

At TOH all clinical environments are supplied with an emergency drug box which is maintained by the pharmacy department. The box contains emergency non-cardiac arrest medications and other resources. The RPNs were aware of the location of the box, but not the contents. The physicians were not aware of the box nor contents. The team was pleasantly surprised to learn the box contains the TOH anaphylaxis algorithm and related medications. This algorithm provides guidelines as to medication and IV fluid administration. In acute anaphylaxis timely administration of epinephrine is paramount to the management of the life-threatening symptoms of angioedema, hypotension and tachycardia. Knowing the location and use of the emergency drug box will assist in this. In addition to this, the RPNs decided to post the algorithm at the nursing station and in each exam room after the ISSs.

**Figure 1:**
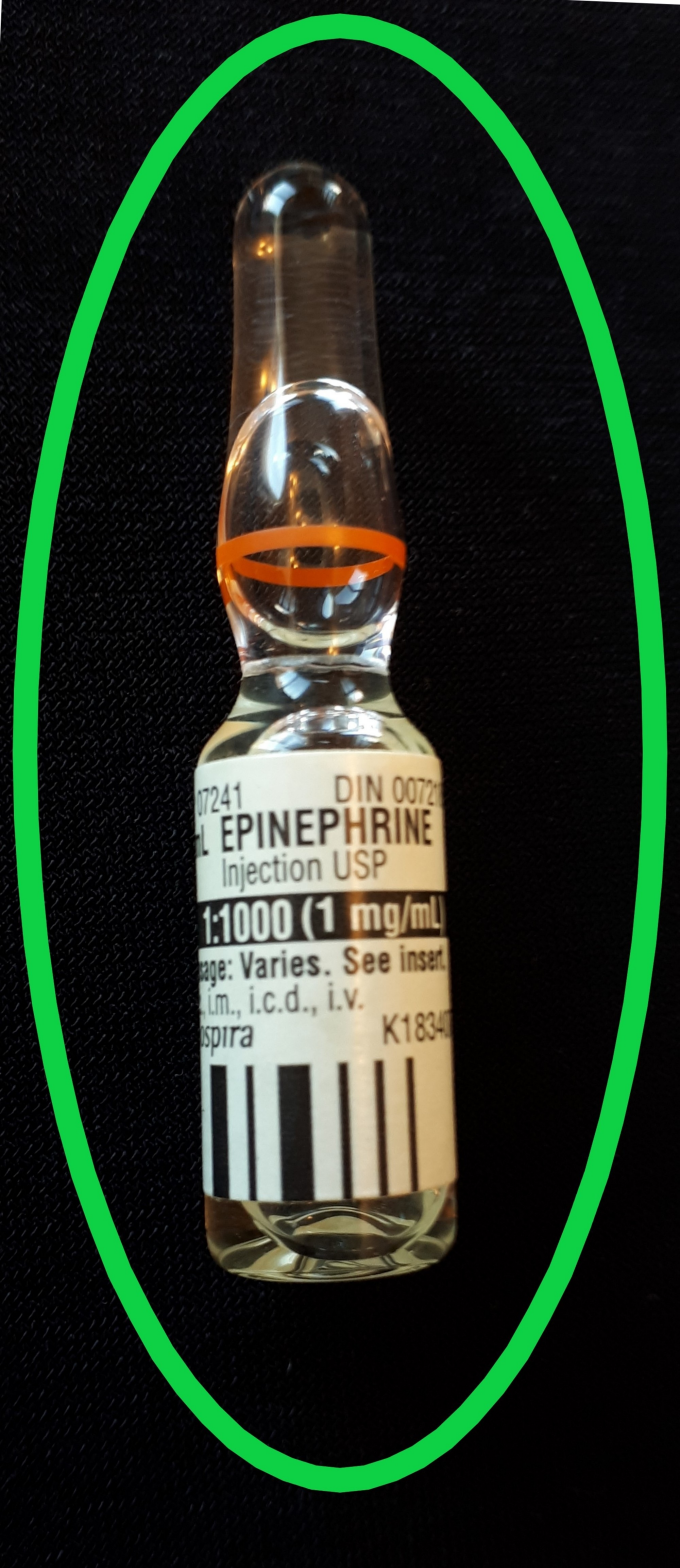
Epinephrine Ampoule 1 mg/1 mL. The safer option for use of epinephrine in anaphylaxis management. mg: milligrams; mL: milliliters.

**Figure 2:**
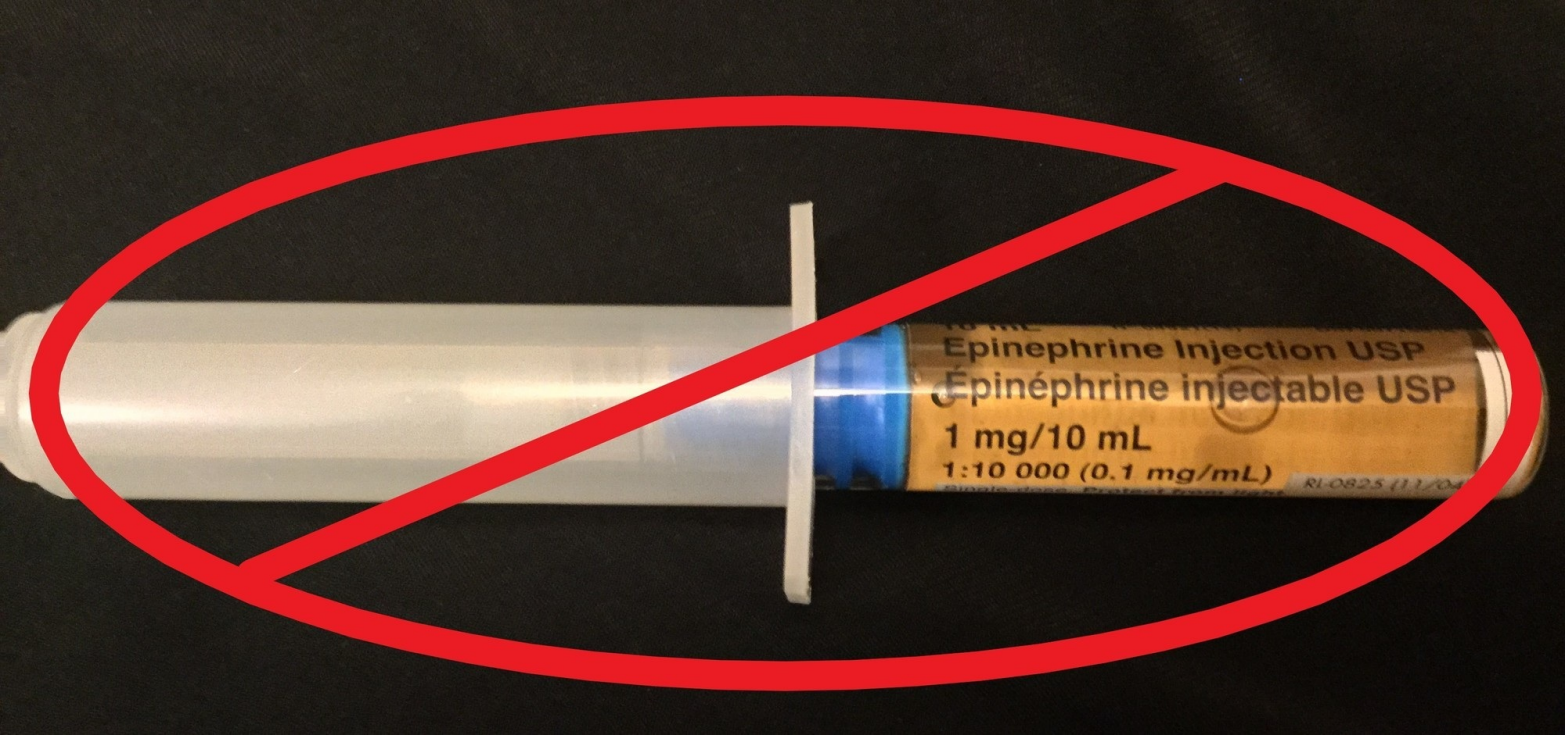
Epinephrine Syringe 1 mg/10 mL. This concentration of epinephrine is appropriate for use in cardiac arrest. There is a greater risk of dosing error when used in anaphylaxis management. mg: milligrams; mL: milliliters.

Discussion occurred around which type of epinephrine packaging to use, specifically the ampoule or pre-filled syringe. The ampoule (Figure 1) is one milligram in one milliliter whereas the pre-filled syringe (Figure 2) is one milligram in 10 milliliters. The pre-filled syringe is intended for use in cardiac arrest where the dose is one milligram. The suggested dose for epinephrine is 300 to 500 micrograms IM or IV in anaphylaxis. The use of the pre-filled one milligram epinephrine syringe in anaphylaxis could inadvertently lead to wrong dosing where the entire syringe is administered resulting in cardiac arrest.

Oxygen administration equipment

Hypoxia is a common sign of anaphylaxis due to the angioedema. Early administration of oxygen can assist in decreasing the extent of the hypoxia. During the ISSs it took an average of three minutes for participants to initiate oxygen administration. This was attributed to the lack of equipment in the treatment room. In one instance the cardiac arrest cart was opened to obtain the equipment. After discussion around the importance of early administration of oxygen in anaphylaxis, the RPNs added oxygen administration supplies to the main treatment room of the clinic.

Crisis resource management

Effective communication in crisis is essential for efficient and successful management in healthcare. The concepts of leadership, follower-ship, delegation and closed loop communication were assessed during the ISSs and discussed in the debrief. Closed loop communication was modeled by the simulation instructors then practiced by participants during the debrief.

Evaluation

At the time of this ISS implementation formal evaluation forms were not being used by the TOH Safe Patient Simulation Program. The healthcare team found the ISSs beneficial to their clinical practice through verbal feedback to the nurse educators and were thankful for the opportunity to participate. The nurse educators noted changes at the behavioral level where RPNs modified their clinical environments including posting of the anaphylaxis algorithm, adding oxygen administration equipment to the treatment room and bundling of IV supplies for emergency situations.

## Conclusions

The initiation of a low frequency high impact ISS of anaphylaxis in the outpatient setting has been discussed. The implementation of this scenario in the OPAT clinic allowed the identification of many LSTs. Through identification of these LSTs solutions were implemented, including the addition of equipment to the practice setting, knowledge of available resources, and use of the appropriate format and concentration of epinephrine in anaphylaxis. Clarity of roles of team members, particularly, the RPN and the opportunity to utilize closed loop communication during the simulation were also key outcomes of this learning opportunity.

## Appendices

Appendix A: Equipment list

·         Manikin

·         Wig

·         T-shirt and pants

·         Treatment chair

·         Primary IV tubing and IV solution bag

·         Secondary IV tubing and mini bag

·         IV pump

·         Various syringes

·         Various needles for IM injection

·         Alcohol swabs

·         Oxygen mask

·         Nasal prongs

·         Vital signs monitor

·         Outpatient medical record

·         Emergency drug box (see appendix B for stocking list)

·         Cardiac arrest cart

Appendix B: Emergency drug box list

**Table 3:** Emergency Drug Box Stocking List. amp: ampoule; meq: milliequivalents; mg: milligrams; mL: milliliters; mg/ml: milligrams per milliliter; syr: syringe.

Medication	Quantity
Acetylsalicylic acid (ASA) 80 mg tablet	2
Calcium Chloride 10% prefilled syr.	2
Dextrose 50% prefilled syr.	2
Diazepam 10 mg/2 mL amp.	5
Diphenhydramine 50 mg/mL vial	2
Ephedrine 50 mg/mL amp.	1
Epinephrine 1:1000 amp.	3
Flumazenil 0.5 mg/5 mL vial	1
Furosemide 40 mg/4 mL amp.	5
Haloperidol 5 mg/mL amp.	5
Hydralazine 20 mg/mL amp.	1
Labetalol 100 mg/20 mL vial	1
Methylprednisolone 125 mg vial	1
Metoprolol 5 mg/5 mL vial	3
Midazolam 10 mg/10 mL vial	1
Naloxone 0.4 mg/2 mL amp.	2
Phenylephrine 10 mg/mL amp.	1
Phenytoin 250 mg/5 mL vial	6
Ranitidine 50 mg/2 mL vial	1
Na Bicarbonate 50 meq/50 mL syr.	2
Verapamil 5 mg/2 mL vial	4
